# Phylogenomic analyses reveal reticulate evolution between *Neomicrocalamus* and *Temochloa* (Poaceae: Bambusoideae)

**DOI:** 10.3389/fpls.2023.1274337

**Published:** 2023-12-04

**Authors:** Zhuo-Yu Cai, Zheng-Yang Niu, You-Yuan Zhang, Yi-Hua Tong, Tien Chinh Vu, Wei Lim Goh, Sarawood Sungkaew, Atchara Teerawatananon, Nian-He Xia

**Affiliations:** ^1^Key Laboratory of Plant Resources, Conservation and Sustainable Utilization/Guangdong Provincial Key Laboratory of Digital Botanical Garden, South China Botanical Garden, Chinese Academy of Sciences, Guangzhou, China; ^2^College of Life Sciences, University of Chinese Academy of Sciences, Beijing, China; ^3^South China National Botanical Garden, Guangzhou, China; ^4^Quality Management Office, Guiyang Vocational and Technical College, Guiyang, China; ^5^State Key Laboratory of Plant Diversity and Specialty Crops, South China Botanical Garden, Chinese Academy of Sciences, Guangzhou, China; ^6^Vietnam National Museum of Nature, Vietnam Academy of Science and Technology, Hanoi, Vietnam; ^7^Graduate University of Science and Technology, Vietnam Academy of Science and Technology, Hanoi, Vietnam; ^8^Faculty of Science, Universiti Tunku Abdul Rahman, Jalan Universiti, Kampar, Perak, Malaysia; ^9^Department of Forest Biology, Faculty of Forestry, Kasetsart University, Bangkok, Thailand; ^10^Natural History Museum of Thailand, National Science Museum, Pathum Thani, Thailand

**Keywords:** single-nucleotide polymorphism, single-copy nuclear gene, phylogenetic incongruence, introgression, incomplete lineage sorting

## Abstract

*Neomicrocalamus* and *Temochloa* are closely related to bamboo genera. However, when considered with newly discovered and morphologically similar material from China and Vietnam, the phylogenetic relationship among these three groups was ambiguous in the analyses based on DNA regions. Here, as a means of investigating the relationships among the three bamboo groups and exploring potential sources of genomic conflicts, we present a phylogenomic examination based on the whole plastome, single-nucleotide polymorphism (SNP), and single-copy nuclear (SCN) gene datasets. Three different phylogenetic hypotheses were found. The inconsistency is attributed to the combination of incomplete lineage sorting and introgression. The origin of newly discovered bamboos is from introgressive hybridization between *Temochloa liliana* (which contributed 80.7% of the genome) and *Neomicrocalamus prainii* (19.3%), indicating that the newly discovered bamboos are closer to *T. liliana* in genetics. The more similar morphology and closer distribution elevation also imply a closer relationship between *Temochloa* and newly discovered bamboos.

## 1 Introduction

The bamboo tribe Bambuseae (Poaceae: Bambusoideae), also known as the paleotropical and neotropical woody bamboos, has a significant role in ecology and socio-economy (Kambale et al., 2022) and is considered a phylogenetically and taxonomically challenging group (Goh et al., 2013; Liu et al., 2020). The cytonuclear discordance is a reflection of reticulate evolution, which makes the paleotropical woody bamboos a phylogenetically recalcitrant group (Liu et al., 2020). The discordance could arise from evolutionary processes, such as hybridization, introgression, and incomplete lineage sorting (ILS) (Smith et al., 2015; Stull et al., 2020). In previous studies, it has been demonstrated that assessing gene flows (hybridization and introgression) and ILS could help us reach a better understanding of closely related taxa (García et al., 2017; Morales-Briones et al., 2018). For paleotropical woody bamboos, some hybrids have been reported among erect bamboos (Goh et al., 2011; Goh et al., 2018); however, hybridization/introgression and ILS, which are presumed as underlying causes for their complex evolutionary history (Goh et al., 2013), have been poorly explored in climbing bamboos.

*Neomicrocalamus* Keng f. is a small genus of climbing or scrambling bamboos, comprising three species and distributed in Bhutan, southwest China, northeast India, and Vietnam (Li and Stapleton, 2006; BPG [Bamboo Phylogeny Group], 2012; Vorontsova et al., 2016). *Neomicrocalamus dongvanensis* T.Q. Nguyen from Vietnam may not belong to this genus as it possesses erect culms (Nguyen, 1991; Stapleton et al., 1997). This genus has often been regarded as related to *Racemobambos* Holttum, another group of climbing or scrambling bamboos distributed in Malesia (Holttum, 1956; Wen, 1986; Stapleton, 1994). However, in the phylogenetic analyses inferred with plastid and nuclear fragments, *Neomicrocalamus* has been shown to be distantly related to *Racemobambos* and sister to *Temochloa* S. Dransf. (Ruiz-Sanchez and Sosa, 2015; Zhou M.Y. et al., 2017).

*Temochloa* is a monotypic genus in Thailand and possibly Laos (Dransfield, 2000; Zhou M.Y. et al., 2017). Up to now, little is known about it. Some crucial morphological characters are still unavailable, such as lodicules, stamens, and fruits. As such, it was not possible to place *Temochloa* in a subtribal classification of Southeast Asian woody bamboos (Wong et al., 2016). Likewise, it was regarded as having an affinity to *Racemobambos* (Dransfield, 2000). However, *Temochloa* possesses some characteristics in common with *Neomicrocalamus* in morphology and biogeography. Both of them are climbing bamboos in limestone areas and have short-necked pachymorph rhizomes and branch complements with many short and subequal branches with an occasional dominant central branch that reiterates and approaches the size of the culm. In phylogenetic analyses based on plastid regions, it was recovered close to the *Bambusa*–*Dendrocalamus*–*Gigantochloa* (BDG) complex and sister to *Neomicrocalamus* (Sungkaew et al., 2009; Kelchner and BPG [Bamboo Phylogeny Group], 2013; Zhou M.Y. et al., 2017). However, substantial conflicting signals were detected in the organelle DNA dataset (Zhou M.Y. et al., 2017).

During fieldwork in North Vietnam, some climbing bamboos came to our attention, but no flowering material was obtained until subsequent exploration in a neighboring area in China (**Table S1**). The Chinese material (collection number: *BH85*) is significant among these newly discovered bamboos as it is the only one containing flowers and fruits. After consulting the literature, potential placements in *Neomicrocalamus* or *Temochloa* were considered for these newly discovered bamboos. These three groups share a number of similar morphological characters, prompting an investigation of their phylogenetic relationships in order to better understand if taxonomic placements may be better justified or improved.

However, the phylogeny based on DNA fragments shows an ambiguous relationship among these three groups. The use of single-nucleotide polymorphism (SNP) datasets has been demonstrated to give a better resolution in bamboo phylogeny (Liu et al., 2020; Guo et al., 2021; Liu et al., 2022). In addition, the multispecies coalescent model used to reconstruct species trees with large numbers of nuclear genes has also much-improved accuracy of phylogenetic inference (McCormack et al., 2009; Smith et al., 2015; Liu et al., 2021). In this study, we aim to 1) examine the relationship among these two genera and the newly discovered bamboos based on the whole plastome, SNP, and single-copy nuclear (SCN) gene datasets with both concatenation and coalescence methods, and 2) assess the potential sources of plastid/nuclear discordance and the gene tree/species tree incongruence.

## 2 Materials and methods

### 2.1 Taxon sampling

For inference of the systematic position, phylogenetic reconstruction was based on plastid loci referred to in the analyses of Zhou M.Y. et al., (2017) and Haevermans et al. (2020). Finally, 23 samples were involved in the reconstruction, among which 15 samples, representing 14 species (including one variety) from nine genera, were downloaded from GenBank. Eight samples were newly sequenced in this study. *Guadua angustifolia* Kunth was set as the outgroup for this analysis.

The eight newly sequenced samples were also used to conduct further analyses on the basis of the complete plastome, SNP, and SCN gene datasets. For the plastome, SNP, and SCN phylogenetic tree reconstruction, another sample was added as the outgroup, *Bonia levigata* (L.C. Chia, H.L. Fung & Y.L. Yang) N.H. Xia. All sample details are provided in **Tables S1** and **S2**.

### 2.2 DNA extraction and sequencing

Total genomic DNA was isolated from silica-dried healthy leaves using the TIANGEN Genomic DNA Extraction Kit (TIANGEN, Beijing, China). DNA samples of concentration meeting the standard (≥1 μg) were randomly sheared into fragments using Covaris M220 (Covaris, Woburn, MA, USA). Fragments of 350 bp were enriched using PCR, and the paired-end (2 × 150 bp) reads were generated in Novogene (Beijing, China) using the NovaSeq 6000 platform. As a result, approximately 20 Gb genome skimming data were generated for each sample. Among species with already published bamboo genome data, *Bonia amplexicaulis* (L.C. Chia, H.L. Fung & Y.L. Yang) N.H. Xia, with a whole genome size of 0.848 Gb (Guo Z.H. et al., 2019), is the species closest to the taxa included in the present investigation (Zhou M.Y. et al., 2017). Hence, the expected sequencing coverage for our samples would be approximately 23.58× (20 Gb/0.848 Gb). For confirmation of the actual coverage, Jellyfish 2.2.3 (Marçais and Kingsford, 2011) and GenomeScope 2.0 (Ranallo-Benavidez et al., 2020) were used to estimate genome size for each newly sequenced sample (including the outgroup).

### 2.3 Plastome assembly, annotation, and plastid locus extraction

All DNA regions were extracted from plastomes using a python script “get_annotated_regions_from_gb.py” (https://github.com/Kinggerm/PersonalUtilities/). Eventually, following the procedures of Zhou M.Y. et al. (2017), 18 plastid loci were selected (*rpl32-trnL*, *trnT-trnL*, *trnL-trnF*, *psbA-trnH*, *rpl16* intron, *rps16-trnQ*, *trnC-rpoB*, *trnD-trnT*, *rps16* intron, *ndhF* [3′ end], *matK*, *atpB-rbcL*, *psbM-petN*, *trnS-trnfM*, *ycf4-cemA*, *trnG-trnT*, *rps15-ndhF*, and *rbcL-psaI*). All plastid loci were concatenated for phylogenetic analysis.

The filtered clean reads were used for *de novo* assembly of complete plastomes using the GetOrganelle v.1.6.2 pipeline (Jin et al., 2020) with six *k*-mer values: 21, 45, 65, 85, 105, and 125. Subsequently, the filtered plastid reads were transferred to Bandage v.0.8.1 (Wick et al., 2015) for the visualization process. Two opposite plastid sequences exported from Bandage were aligned with the reference sequence *Bonia saxatilis* (L.C. Chia, H.L. Fung & Y.L. Yang) N.H. Xia (GenBank accession No. MK679779), and the one matching the reference was annotated using PGA (Qu et al., 2019). Finally, plastomes were manually corrected in Geneious v.9.1.4 (Kearse et al., 2012).

### 2.4 SNP dataset construction

The latest high-quality genome sequence of *Dendrocalamus latiflorus* Munro (Zheng et al., 2022) was selected as the chromosome-level reference to build an index using SAMtools v.1.9 (Danecek et al., 2021) and Picard v.2.27.3 (Broad, 2019). After the filtration of low-quality data, clean reads were processed with the removal of duplicates using Fastuniq v.1.1 (Xu et al., 2012). Newly filtered paired reads were aligned to the reference using Bowtie2 v.2.4.4 (Langmead and Salzberg, 2012) with the minimum acceptable alignment score set as L, 0.3, 0.3. SAMtools was invoked to sort out alignments in binary alignment format (BAM) files. Picard was used to remove duplicates again with the function “MarkDuplicates”. GATK v.4.2.2.0 (van der Auwera and O'Connor, 2020) was used to anchor variable sites including SNP and InDel in the genomic variant call format (GVCF) file by the joint-calling method “HaplotypeCaller” with the minimum assembly region size as 10 and *k*-mer from 10 to 25. After completion of variant calling, the tool “CombineGVCFs” in GATK was performed to combine all the GVCF files. The tool “GenotypeGVCFs” was then used to identify all the joint-called variants. After that, filtration of low-quality SNPs was conducted with the tool “VariantFiltration” with the following parameters: QD < 2.0, MQ < 40.0, FS > 60.0, SOR > 3.0, MQRankSum < −12.5, and ReadPosRankSum < −8.0. Then, the tool “SelectVariants” was run to extract raw SNPs. After extraction, plink v.1.90b4.6 (Purcell et al., 2007) was used to filter low-quality SNPs with the parameter “geno” set as 0.1 and “maf” set as 0.01. Filtered variants were then pruned with the parameter “indep-pairwise” set as 50, 10, and 0.2, representing its window size, a variant count to shift the window, and pairwise r2 threshold for SNPs, respectively. Finally, a clean SNP dataset was generated, and the GVCF file was transferred to a PHYLIP file for phylogenetic analysis using the python script “vcf2phylip.py” (Ortiz, 2019).

### 2.5 SCN dataset construction

The protein-coding sequences of five previously published bamboo genomes, namely, *Phyllostachys edulis* (Carrière) J. Houz. (Zhao et al., 2018), *B. amplexicaulis*, *G. angustifolia* Kunth, *Olyra latifolia* L., and *Raddia guianensis* (Brongn.) Hitchc. (Guo Z.H. et al., 2019), were used to identify conserved orthologous genes based on the Liliopsida and Poales datasets of BUSCO v.4.1.3 (Manni et al., 2021). Then, the selected orthologous genes of these five bamboo genomes were used to further identify the single-copy nuclear genes using OrthoFinder v 2.4.0 (Emms and Kelly, 2019). A total of 443 genes were generated and set as the reference gene dataset. The filtered reads were mapped to the reference and assembled using the software HybPiper v 1.3 (Johnson et al., 2016) with default parameters. Finally, following the previous study (Zhou et al., 2022), these assembled genes, which are longer than 200 bp, were used in the subsequent analyses.

### 2.6 Phylogenetic analysis

Each dataset (plastid locus, whole plastome, SNP, single SCN gene, and concatenated SCN genes) was aligned using MAFFT v.7.450 (Katoh and Standley, 2013). For each data matrix, both maximum likelihood (ML) and Bayesian inference (BI) analyses were conducted. Maximum likelihood analysis was carried out in RAxML-HPC v.8.2.10 (Stamatakis, 2014), with a GTRGAMMA nucleotide substitution model and 1,000 rapid bootstrap replicates. Bayesian inference analysis was carried out using MrBayes v.3.2.7 (Ronquist et al., 2012). The HKY+I+G model, deduced using MrModeltest2 v.2.4 (Nylander, 2004) under the Akaike information criterion (AIC) (Posada and Buckley, 2004), was selected for the plastid locus matrix, as well as the plastome matrix, the GTR model was selected for the SNP matrix, and the GTR+I+G model was selected for the SCN gene concatenated matrix. At least 30,000,000 generations were run to ensure that the average standard deviation of split frequencies was lower than 0.01, with the sampling frequency set as 100 generations. The first 25% of sampled trees were discarded as burn-in.

To infer species trees, two different approaches were used. The first method was conducted using ASTRAL-III v.5.7.8 (Zhang et al., 2018). Individual SCN gene trees were estimated using RAxML-HPC v.8.2.10 (Stamatakis, 2014) with a GTRGAMMA model and 500 rapid bootstrap replicates. Branches of each gene tree with support lower than 50% were removed to improve the accuracy of species tree inference. Then, individual gene trees and their bootstrap replicates were used to estimate species trees with 500 coalescent bootstrap replicates. SVDquartets v.1.0 (Chifman and Kubatko, 2014) implemented in PAUP v.4.0a169 (Swofford, 2003) was the second method that utilizes the concatenated SCN gene matrix to infer the species tree. The clade support was assessed using 1,000 bootstrap replicates.

The final phylogenetic results were visualized using FigTree v.1.4.4 (http://tree.bio.ed.ac.uk/software/figtree/).

### 2.7 Network analysis

In order to investigate potential conflicting signals within the plastid locus matrix, the Neighbor-Net algorithm based on uncorrected *P*-distances was performed with SplitsTree4 v.4.18.1 (Huson and Bryant, 2006).

### 2.8 Coalescent simulation

To test if the discordance between plastome tree and nuclear trees could be explained by ILS alone, we conducted coalescent simulations following previous studies (García et al., 2017; Morales-Briones et al., 2018; Zhou et al., 2022). The ASTRAL-III tree was chosen as a guide tree for the gene tree simulation using DENDROPY v3.12.1 (Sukumaran and Holder, 2010). To simulate plastid gene trees, branch lengths of the ASTRAL-III tree were scaled by 12 to account for organellar inheritance as paleotropical woody bamboos are hexaploidy, and the effective population size of the plastome is generally expected to be one-twelfth that of the nuclear genome given the assumptions of equal sex ratios, haploidy (homoplasmic), and uniparental inheritance (McCauley, 1994; Stull et al., 2020). Finally, 1,000 gene trees were simulated under the coalescent model. The clade frequencies of simulated trees were summarized on the plastome tree inferred using RAxML with PHYPARTS (Smith et al., 2015). In a scenario of ILS alone, the clades in the empirical plastome tree should be present in the simulated trees with high frequency. If hybridization exists, the clades of the empirical plastome trees should be absent or at very low frequency in the simulated trees (García et al., 2017; Morales-Briones et al., 2018; Zhou et al., 2022).

### 2.9 Gene flow analyses

To detect potential gene flows between lineages, the D-statistic test was performed in Dsuite v.0.5 (Malinsky et al., 2021). Each lineage was represented by all samples of each clade. *B. levigata* was treated as the outgroup. Z-scores greater than three are generally interpreted as strong evidence of gene flow; otherwise, ILS cannot be excluded as an explanation for ancient polymorphisms at maximum probability (Eaton and Ree, 2013; Ye et al., 2021).

To further explore the reticulate evolutionary history, SnaQ (Solís-Lemus and Ané, 2016) implemented in PhyloNetworks v.0.12.0 (Solís-Lemus et al., 2017) was used to infer species networks. The input was SCN gene trees estimated using RAxML. Analyses allowing for 0–4 hybridization (*h*) events were performed using 10 independent runs. The best network was selected when pseudolikelihood scores reached a nearly constant level.

## 3 Results

### 3.1 Basic features of three datasets

For the newly sequenced samples, the estimated genome size and the coverage ranged from 747.32 Mb to 927.72 Mb and from 21.56× to 26.76×, respectively (**Table S3**).

The dataset for the combined and aligned matrix of 18 plastid loci across 23 samples consisted of 18,484 bp. A total of 787 (4.26%) variable sites were found, of which 301 (1.63%) were parsimony informative sites and 486 (2.63%) were singleton variable sites. Missing data accounted for 5.5% of the entire matrix.

The plastome size ranged from 138,292 bp to 139,510 bp. The alignment of the plastome of nine samples comprised 140,288 bp, characterized using only 976 (0.69%) variable sites, including 327 (0.23%) parsimony informative sites and 649 (0.46%) singleton variable sites. Missing data accounted for only 0.7% of the entire matrix.

For the SNP data, the matrix was 1,651 bp in total length with 1,490 (90.25%) variable sites, including 279 (16.90%) parsimony informative sites and 1,211 (73.35%) singleton variable sites. The SNP data matrix did not have missing data.

A total of 358 SCN genes were obtained for each sample. The concatenated matrix for 358 SCN genes consisted of 618,938 bp, featuring 48,971 (7.91%) variable sites, of which 21,237 (3.43%) were parsimony informative sites and 27,734 (4.48%) were singleton variable sites. For the whole matrix, the missing data were 5.3%.

### 3.2 Systematic position inferred using 18 plastid loci

**Figure 1:**
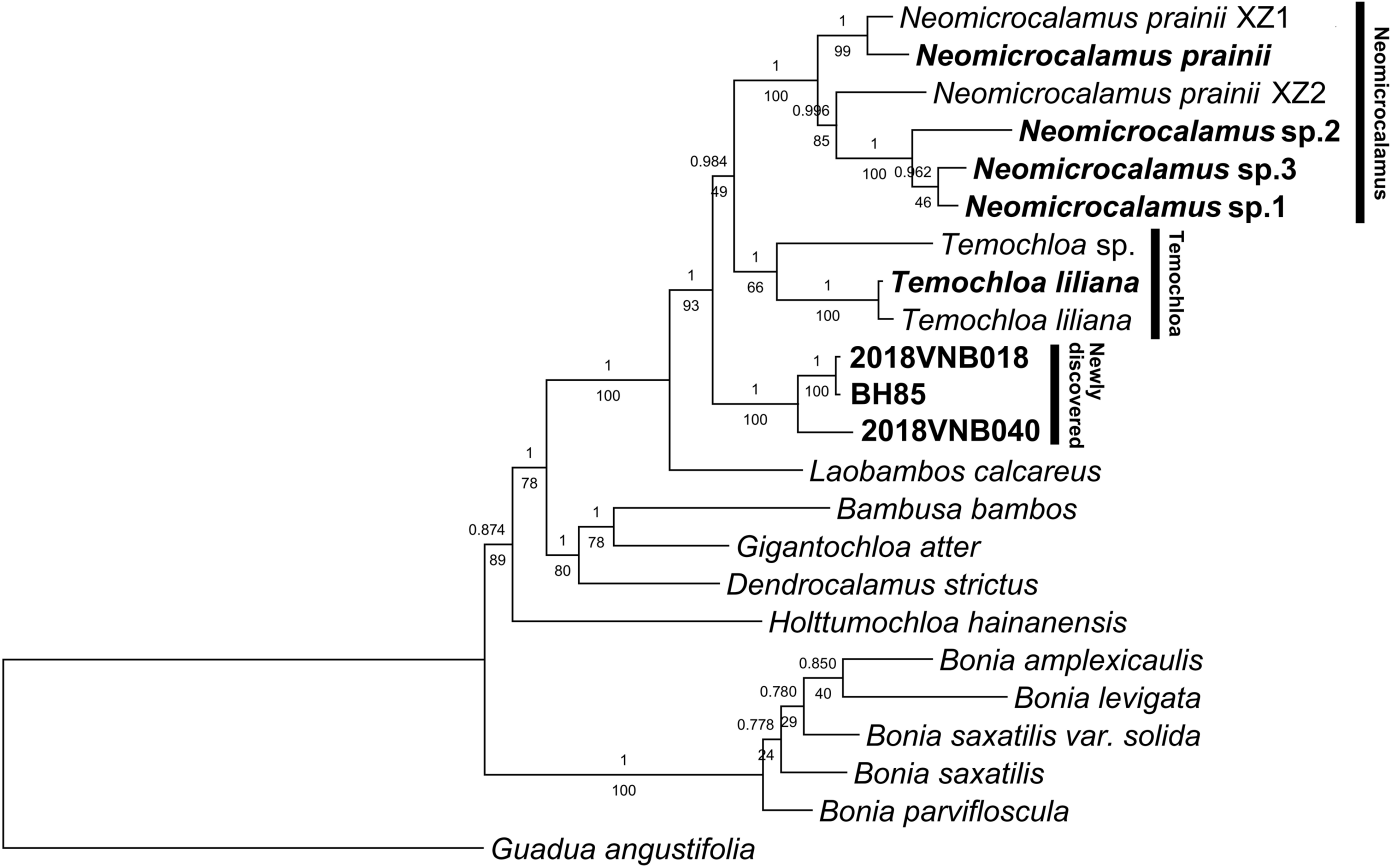
Majority rule (50%) consensus phylogenetic tree based on a combined plastid dataset of 18 loci. Both Bayesian inference (BI) and maximum likelihood (ML) analyses were conducted. Numbers above and below branches indicate Bayesian inference posterior probability (PP) and bootstrap (BS), respectively. *BH85*, *2018VNB018*, and *2018VNB040* represent the newly discovered bamboos; these and other accessions freshly obtained for this analysis are indicated in bold.

Inference of phylogenetic relationships using plastid loci conducted with different methods (ML and BI) yielded identical topologies but different support values for some nodes (**Figure 1**). Newly sequenced *Neomicrocalamus* samples clustered with published *Neomicrocalamus* samples (**Figure 1**), forming a well-supported *Neomicrocalamus* clade (Bayesian inference posterior probability [PP]/bootstrap [BS], 1/100). The two *Temochloa liliana* S. Dransf. samples were recovered together with PP/BS 1/100 support and sister to *Temochloa* sp. (PP/BS 1/66), together making up the *Temochloa* clade. The three newly discovered bamboo samples formed a clade with PP/BS 1/100 support and, together with *Neomicrocalamus* spp. and *Temochloa* spp., represent a monophyletic lineage with high support (PP/BS 1/93). However, the relationship among the three clades of this lineage was unresolved as one of the pivotal nodes was not fully supported (PP/BS 0.984/49).

The network analysis revealed a splitting pattern similar to the phylogenetic tree topology, in which *Neomicrocalamus*, *Temochloa*, and the newly discovered bamboo clusters were connected by many parallel short edges, indicating character conflicts among these three clades (**Figure S1**).

### 3.3 Relationship revealed with plastome, SNP, and SCN

As with the plastid locus dataset, ML and BI analysis methods produced identical topologies. However, three backbone topologies were revealed with different datasets and methods: Topology A (recovered using plastome), Topology B (SNP and ASTRAL-III), and Topology C (concatenated SCN and SVDquartets).

**Figure 2:**
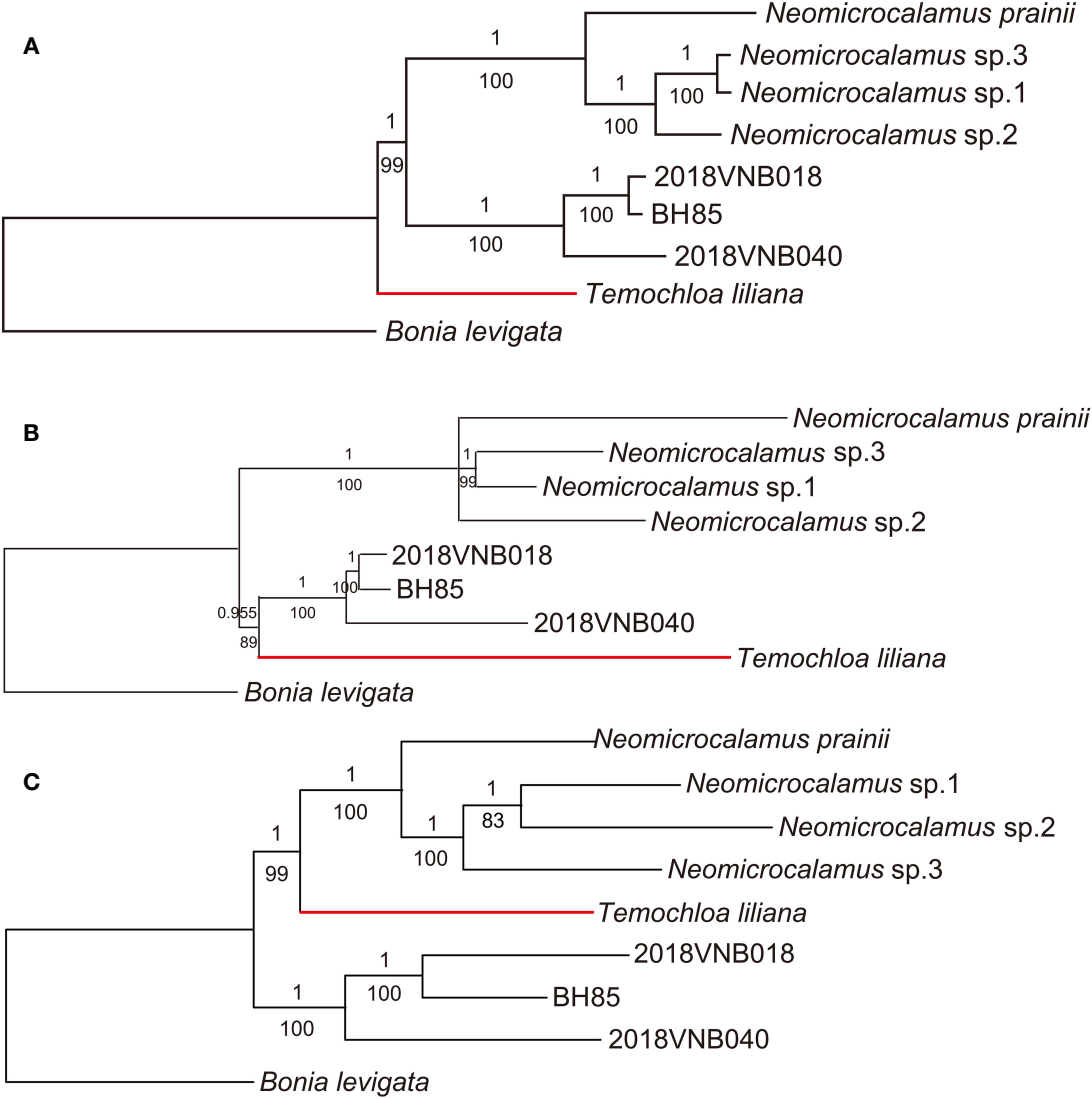
Phylogenetic trees of three groups of climbing bamboos based on the whole plastome **(A)**, the single-nucleotide polymorphism (SNP) **(B)**, and the concatenated single-copy nuclear (SCN) genes **(C)**. Both Bayesian inference (BI) and maximum likelihood (ML) analyses were conducted for each dataset. Numbers above and below branches indicate Bayesian inference posterior probability (PP) and bootstrap (BS), respectively. *BH85*, *2018VNB018*, and *2018VNB040* represent the newly discovered bamboos. The red branches indicate the position of *Temochloa*.

In the well-resolved plastome phylogeny, with almost every node being fully supported (PP/BS 1/100), the newly discovered bamboo clade was sister to the *Neomicrocalamus* clade, and the *Temochloa* clade was in turn sister to both of them (**Figure 2A**). The SNP phylogeny was also well-resolved except for the relationship within the *Neomicrocalamus* clade. The clade comprising the newly discovered bamboos aligned with the *Temochloa* clade as a monophyletic lineage with PP/BS 0.955/89 support, which was sister to the *Neomicrocalamus* clade (**Figure 2B**). In the phylogenetic tree inferred with the concatenated SCN genes, the *Temochloa* clade and the *Neomicrocalamus* clade together form a well-supported (PP/BS 1/99) monophyletic lineage. The newly discovered bamboo clade is sister to them (**Figure 2C**).

**Figure 3:**
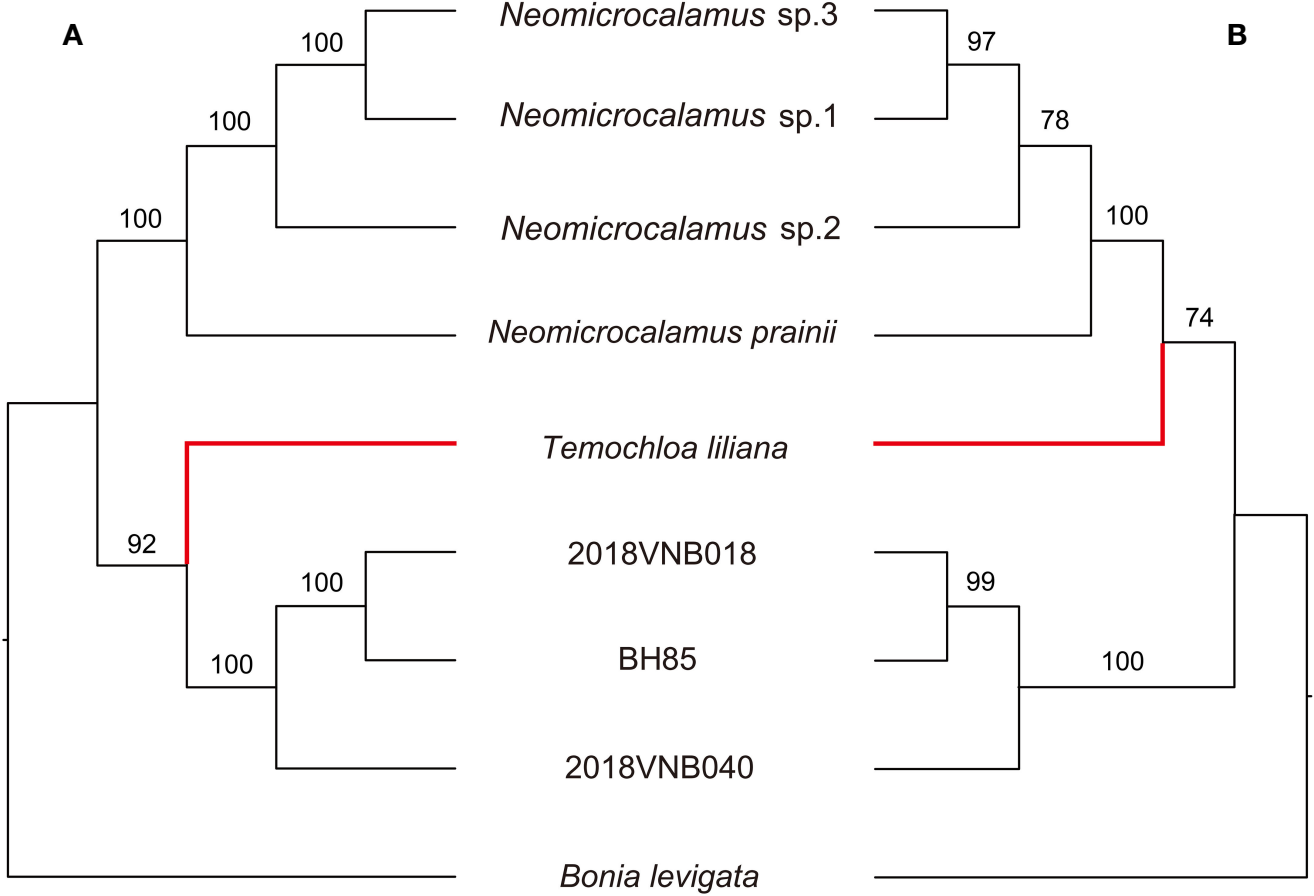
Tanglegram comparing the species trees inferred in ASTRAL-III **(A)** and SVDquartets **(B)**. Numbers above branches indicate bootstrap (BS). *BH85*, *2018VNB018*, and *2018VNB040* represent the newly discovered bamboos. The red branches indicate the position of *Temochloa*.

In the species trees, the backbone topology recovered using SVDquartets is the same as the concatenated SCN gene tree. However, the support value for the sister group relationship of the *Temochloa* clade and the *Neomicrocalamus* clade is not high enough (BS 74 < 75) (**Figure 3**). However, the ASTRAL-III found the *Temochloa* clade is sister to the newly discovered bamboo clade with good support (BS 92), in agreement with the SNP phylogeny.

Furthermore, the relationship within the newly discovered bamboo clade is always identical among the five phylogenies. Although *Neomicrocalamus prainii* (Gamble) Keng f. is always sister to the other three *Neomicrocalamus* species, the position of *Neomicrocalamus* sp.2 is varied. In most of the phylogenies, *Neomicrocalamus* sp.1 is sister to *Neomicrocalamus* sp.3; however, in the concatenated SCN gene phylogeny, the position of *Neomicrocalamus* sp.3 is replaced by *Neomicrocalamus* sp.2.

### 3.4 Assessment ILS and gene flow

**Figure 4:**
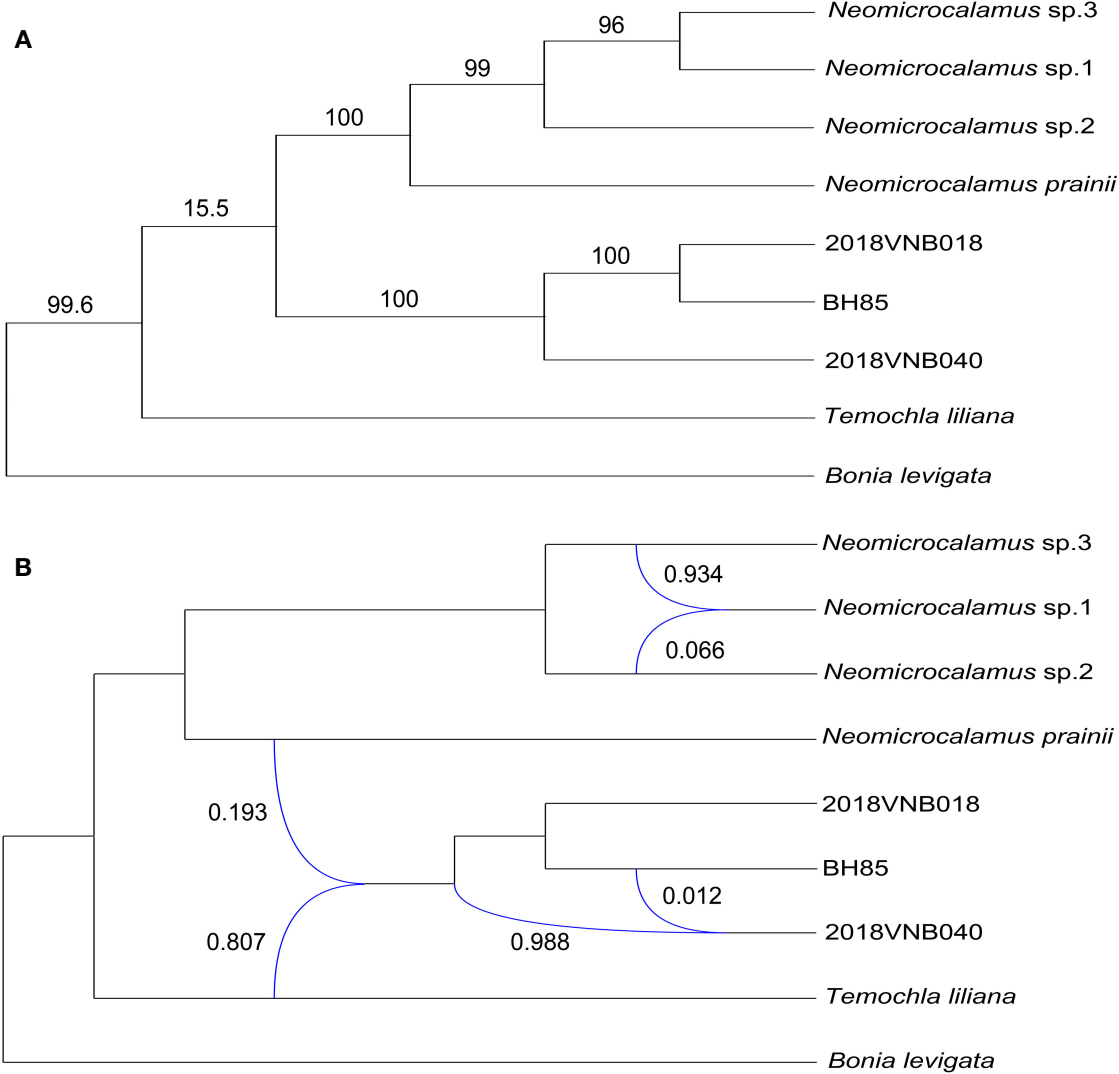
**(A)** Plastome phylogeny inferred with maximum likelihood; numbers above branches represent clade frequencies of the simulated gene trees. **(B)** Inference of gene flows; blue lines indicate hybrid edges, and numbers near blue lines are vectors of inheritance probabilities (γ).

The plastid gene trees produced by the coalescent simulations resembled the empirical plastome tree. After summarizing simulated plastid gene trees onto the empirical plastome tree, most clade frequencies were at or near 100%, except for the clade of sister groups, the *Neomicrocalamus* group, and the newly discovered bamboo group, which is only 15.5% (**Figure 4**).

From the D-statistic test, the Z-score was 1.63 lower than 3, which indicates both gene flows and ILS could be because of ancient polymorphisms (**Table S4**). However, the p-value (0.101 > 0.05) suggests that the result is only for reference.

Finally, three hybridization/introgression events were inferred (**Figure 4** and **Table S5**). The results show that the newly discovered bamboo group inherited 80.7% of its genome from *T. liliana* and inherited 19.3% from *N. prainii* (**Figure 4**). The other two events are located within the *Neomicrocalamus* group and the newly discovered bamboo group, respectively. Both of them show largely unequal (ca. 95% vs. ca. 5%) gene flows between parents.

## 4 Discussion

In the previous phylogenetic analyses, *Neomicrocalamus* and *Temochloa* were revealed as closely related genera (sister groups) (Zhou M.Y. et al., 2017), which is also supported by some morphological and biogeographic evidence, such as short-necked pachymorph rhizomes and climbing culms endemic to limestone areas. After adding more accessions into the matrix of 18 plastid loci, the relationships among the main groups were congruent with those found in a former study (Zhou M.Y. et al., 2017), and the close relationship of these two genera is further confirmed, but these two genera together with the third bamboo group formed a three-clade polytomy (**Figure 1**) accompanied with substantial conflicting signals (**Figure S1**).

In the further phylogenetic analyses, three backbone topologies were recovered: Topology A (recovered by plastome), Topology B (SNP and ASTRAL-III), and Topology C (concatenated SCN and SVDquartets). The differences among topologies show the plastid/nuclear discordance and the gene tree/species tree incongruence. For the incongruence between two species trees ASTRAL-III and SVDquartets, we view the ASTRAL-III topology as more likely to be accurate as in the SVDquartets topology, the sister relationship between *Temochloa* clade and *Neomicrocalamus* clade is uncertain (low BS).

The cytonuclear discordance has been widely reported in plants (Mugrabi De Kuppler et al., 2015; Scheunert and Heubl, 2017; Soltis et al., 2019), as well as in Bambusoideae (Zhang et al., 2012; Wang et al., 2017; Guo C. et al., 2019). Our results of the coalescent simulation suggest that ILS was at play in the speciation of these taxa in this study, causing the cytonuclear discordance (Stull et al., 2020). Furthermore, the D-statistic test results (Z-score value < 3) also could not reject the existence of ILS (Eaton and Ree, 2013). The rapid evolutionary radiation and the long generation time of woody bamboos (Janzen, 1976; Guo Z.H. et al., 2019) are expected to maintain much of their ancestral polymorphism implying opportunities for extensive ILS (Pease and Hahn, 2015; Zhou Y. et al., 2017). Moreover, the hybridization/introgression events identified in this case also could be one of the reasons for the phylogeny incongruence (Linder and Rieseberg, 2004; Folk et al., 2017). In view of the largely unequal proportion of genes contributed by each parental lineage inferred by PhyloNetworks, the gene flows detected here are better interpreted as introgression (Solís-Lemus et al., 2017). The gene flow analyses suggest that the newly discovered bamboos originated from the introgressive hybridization between *T. liliana* and *N. prainii*, with *T. liliana* contributing 80.7% of the genome. In other words, genetically, the newly discovered bamboos are closer to *T. liliana*.

The similarity between the newly discovered bamboos and *Temochloa* is also indicated by another two aspects. In the biogeography, the newly discovered bamboos share the same distribution elevation with *Temochloa* 50–250 m, rarely reaching 700 m, whereas the *Neomicrocalamus* taxa are hitherto only found above 1,000 m (Dransfield, 2000; Li and Stapleton, 2006). This somehow interprets why *T. liliana* contributed a much higher genome component to the newly discovered bamboos, as gene flow is expected to happen more commonly among neighboring populations than distantly located populations (Petit and Excoffier, 2009; Zhou Y. et al., 2017). Morphologically, the newly discovered bamboos and *Temochloa* have nearly circular primary branch buds and shallowly grooved culm leaf sheaths, which are distinguished from the lanceolate buds and plane culm leaf sheaths of *Neomicrocalamus* (Dransfield, 2000; Li and Stapleton, 2006). On balance, the newly discovered bamboos have a closer relationship with *Temochloa*.

The phylogenetic network would help visualize clearer evolutionary relationships when reticulation events are involved (Morales-Briones et al., 2018). Empirically, the bifurcating phylogenetic tree inferred with SNP is more consistent with the evidence from morphology conventionally used to form classifications and genus circumscriptions for bamboos (Liu et al., 2020; Guo et al., 2021; Liu et al., 2022). In view of the limitation of bifurcating phylogenetic trees, the monophyletic group comprising *Temochloa* and the newly discovered bamboos, recovered in the SNP tree and ASTRAL-III species tree, seems to be the best classification for these two groups; as aforementioned, they are more closely related. However, overall, these three groups are closely related phylogenetically and morphologically. Therefore, for a more comprehensive understanding of their relationships, more evidence from all aspects should be investigated, such as the stamen of *Temochloa*, which is still unknown (Dransfield, 2000).

## Data availability statement

The data presented in the study are deposited in the NCBI repository, accession number: OQ791227 OQ791228 OQ791225 OQ791224 OQ791221 OQ791226 OQ791222 OQ791223 OQ791229 (more details in **Table S1**). And it is also included in the **Supplementary Material**.

## Ethics statement

All plant species used in the present study are not specially protected. The collections were permitted by the National Nature Reserve of Yarlung Tsangpo Grand Canyon, China; the Ba Be National Park, Vietnam; and the Klong Phanom National Park, Thailand. All plant experiments were conducted in accordance with relevant institutional, national, and international guidelines and legislation.

## Author contributions

NX: Conceptualization, Funding acquisition, Writing – review & editing. ZC: Formal analysis, Investigation, Methodology, Writing – original draft, Writing – review & editing. ZN: Formal analysis, Methodology, Software, Writing – review & editing. YZ: Investigation, Writing – review & editing. YT: Investigation, Writing – review & editing. TV: Investigation, Writing – review & editing. WG: Formal analysis, Writing – review & editing. SS: Investigation, Writing – review & editing. AT: Investigation, Writing – review & editing.
