# Supplementary material for: Phylogenomic analyses reveal reticulate evolution between Neomicrocalamus and Temochloa (Poaceae: Bambusoideae)
Source: Front Plant Sci. 2023 Dec 4;14:1274337. doi: 10.3389/fpls.2023.1274337 (PMC10726129; doi:10.3389/fpls.2023.1274337)
Supplement: Supplementary file 2 [file DataSheet_2.docx]

**Table S1.** Sample information of complete chloroplast genome, SNP data and SCN data. The first 8 samples were also used for the reconstruction of the phylogenetic tree based on plastid loci.

| **Taxon** | **Voucher** | **Locality** | **GenBank accession** |
| --- | --- | --- | --- |
| *Neomicrocalamus* sp.1 | CZY107 (IBSC) | Yunnan, China | OQ791227 |
| *Neomicrocalamus* sp.2 | CZY142 (IBSC) | Yunnan, China | OQ791228 |
| *Neomicrocalamus* sp.3 | TYH2100 (IBSC) | Guangxi, China | OQ791225 |
| *Neomicrocalamus prainii* (Gamble) Keng f. | TYH2350 (IBSC) | Xizang, China | OQ791224 |
| *Temochloa liliana* S. Dransf. | Sungkaew s.n. (KUFF*) | Surat Thani, Thailand | OQ791221 |
| The newly discovered bamboo (unnamed) | BH85 (IBSC) | Guangxi,  China | OQ791226 |
| The newly discovered bamboo (unnamed) | 2018VNB018 (IBSC) | Bac Kan, Vietnam | OQ791222 |
| The newly discovered bamboo (unnamed) | 2018VNB040 (IBSC) | Ha Giang, Vietnam | OQ791223 |
| **Outgroup** | | |  |
| *Bonia levigata* (L.C. Chia, H.L. Fung & Y.L. Yang) N.H. Xia | NH050 (IBSC) | Guangdong, China | OQ791229 |

*KUFF refers to the Herbarium of the Faculty of Forestry, Kasetsart University, Bangkok, Thailand.

**Table S2.** Sample information of plastid loci from GenBank. GenBank accessions are given in the following sequence: *rpl32-trnL*, *trnT-trnL*, *ycf4-cemA*, *trnG-trnT*, *rps15-ndhF*, *rbcL-psaI*, *trnL-trnF*, *psbA-trnH*, *rps16* intron*, rps16-trnQ*, *trnC-rpoB*, *trnD-trnT*, *rpl16* intron*, ndhF* (3′ end)*, matK, atpB-rbcL, psbM-petN* and *trnS-trnfM*. Dash (–) indicates that there are no available sequences in GenBank.

| **Taxon** | **Voucher** | **Locality** | **GenBank accession** |
| --- | --- | --- | --- |
| *Bambusa bambos* (L.) Voss | DZL200202 (KUN) | Ghana | KT227896, KT226359, KT226729, KT227193, KT226481, KT226603, KT226850, KT227656, KT227075, KT227778, KT228133, KT227542, KT228020, KT227307, KT226117, KT227421, KT226239, – |
| *Bonia amplexicaulis* (L.C. Chia, H.L. Fung & Y.L. Yang) N.H. Xia | Zhang Y.X. 12329 (KUN) | Guangxi, China | KT227924, KT226387, KT226757, KT227221, KT226509, KT226631, KT226878, KT227684, KT227103, KT227806, KT228160, KT227570, KT228048, KT227335, KT226145, KT227449, KT226267, KT226991 |
| *Bonia levigata* (L.C. Chia, H.L. Fung & Y.L. Yang) N.H. Xia | Zhang Y.X. 06076 (KUN) | Hainan, China | KT227925, KT226388, KT226758, KT227222, –, KT226632, KT226879, KT227685, KT227104, KT227807, KT228161, KT227571, KT228049, KT227336, KT226146, KT227450, KT226268, KT226992 |
| *Bonia parvifloscula* (W.T. Lin) N.H. Xia | Zhang Z01 (unknown) | Yunnan, China | KT227926, KT226389, KT226759, KT227223, –, KT226633, KT226880, KT227686, KT227105, KT227808, KT228162, KT227572, KT228050, KT227337, KT226147, KT227451, KT226269, KT226993 |
| *Bonia saxatilis* (L.C. Chia, H.L. Fung & Y.L. Yang) N.H. Xia | Zhang Y.X. 12327 (KUN) | Guangxi, China | KT227927, KT226390, KT226760, KT227224, –, KT226634, KT226881, KT227687, KT227106, KT227809, KT228163, KT227573, KT228051, KT227338, KT226148, KT227452, KT226270, KT226994 |
| *Bonia saxatilis* var. *solida* (C.D. Chu & C.S. Chao) D.Z. Li | Zhang Y.X. 12331 (KUN) | Yunnan, China | KT227928, KT226391, KT226761, KT227225, –, KT226635, KT226882, KT227688, KT227107, KT227810, KT228164, KT227574, KT228052, KT227339, KT226149, KT227453, KT226271, KT226995 |
| *Dendrocalamus strictus* (Roxb.) Nees | Zhou M.Y. 018 (KUN) | Guangdong, China | KT227962, KT226425, KT226795, KT227257, KT226544, KT226669, KT226915, KT227722, KT227141, KT227843, KT228198, KT227607, KT228085, KT227372, KT226183, KT227487, KT226305, KT227026 |
| *Gigantochloa atter* (Hassk.) Kurz | AUS 200901 (KUN) | Australia | KT227970, KT226433, KT226803, KT227265, KT226552, KT226677, KT226923, KT227730, KT227149, KT227851, KT228206, KT227615, KT228093, KT227380, KT226191, KT227495, KT226313, KT227033 |
| *Holttumochloa hainanensis* M.Y. Zhou & D.Z. Li | Zhou M.Y. 033 (KUN) | Hainan, China, | KT227988, KT226451, KT226820, KT227279, KT226570, KT226695, KT226937, KT227747, KT227164, KT227866, KT228221, KT227628, KT228106, KT227393, KT226208, KT227512, KT226329, KT227047 |
| *Laobambos calcareus* Haev., Lamxay & D.Z. Li | Li & Zhang 13cs6294-14 (KUN) | Khamoua-  ne, Laos | KT227895, KT226358, KT226728, KT227192, KT226480, KT226602, KT226849, KT227655, KT227074, KT227777, KT228132, KT227541, KT228019, KT227306, KT226116, KT227420, KT226238, KT226964 |
| *Neomicrocalamus prainii* (Gamble) P.C. Keng XZ1 | LL 07236 (KUN) | Xizang, China | KT227998, KT226461, KT226829, KT227288, KT226580, KT226705,  KT226947, KT227757, KT227174, KT227876, KT228231, KT227638, KT228116, KT227403, KT226218, KT227522, KT226339, KT227057 |
| *Neomicrocalamus prainii* (Gamble) P.C. Keng XZ2 | MPF 10060 (KUN) | Xizang, China | KT227997, KT226460, KT226828, KT227287, KT226579, KT226704, KT226946, KT227756, KT227173, KT227875, KT228230, KT227637, KT228115, KT227402, KT226217, KT227521, KT226338, KT227056 |
| *Temochloa liliana* S. Dransf. | (not stated) | (not stated) | –, KC020563.1, –, –, –, –, EU434076.1, –, EU434204.1, KF365125.1, KF365172.1, KC020543.1, KC020528.1, KF365011.1, EU434268.1, EU434140.1, KF365061.1, KF365225.1 |
| *Temochloa* sp. | THLAO 005 (MNHN) | Khamoua-ne, Laos | KT228017, KT226478, KT226847, KT227304, KT226600, KT226726, KT226962, KT227775, KT227190, KT227893, KT228247, KT227653, KT228130, KT227418, KT226236, KT227539, KT226356, KT227072; |
| **Outgroup** | | | |
| *Guadua angustifolia* Kunth | Zhang Y.X. 12325 (KUN) | Taiwan, China | KT227984, KT226447, KT226816, KT227277, KT226566, KT226691, KT226935, KT227743, KT227161, KT227863, KT228219, KT227626, KT228104, KT227391, KT226204, KT227508, KT226325, KT227045 |

**Table S3** Estimated genome size, sequencing coverage and assembly coverage of plastome.

| Samples | Genome size (Mb) | Sequencing coverage | Assembly coverage of plastomes |
| --- | --- | --- | --- |
| CZY107 | 927.72 | 21.56× | 364× |
| CZY142 | 921.89 | 21.69× | 305× |
| TYH2100 | 915.50 | 21.85× | 226× |
| TYH2350 | 854.79 | 23.40× | 533× |
| Sungkaew s.n. | 747.32 | 26.76× | 81× |
| BH85 | 900.56 | 22.21× | 90× |
| 2018VNB018 | 892.94 | 22.40× | 173× |
| 2018VNB040 | 883.97 | 22.63× | 158× |
| NH050 | 856.10 | 23.36× | 532× |

**Table S4** D-statistic test result of three groups of scrambling bamboo.

| **P1** | **P2** | **P3** | **D-statistic** | **Z-score** | **P-value** |
| --- | --- | --- | --- | --- | --- |
| The newly discovered bamboos | *Temochloa* | *Neomicrocalamus* | 0.261657 | 1.63953 | 0.101102 |

**Table S5** The negative log psuedolikelihood for phylogenetic network estimation

| hybridization event (*h*) | 0 | 1 | 2 | 3 | 4 |
| --- | --- | --- | --- | --- | --- |
|  | 80.29 | 45.70 | 43.88 | **0.00** | 0.00 |

**
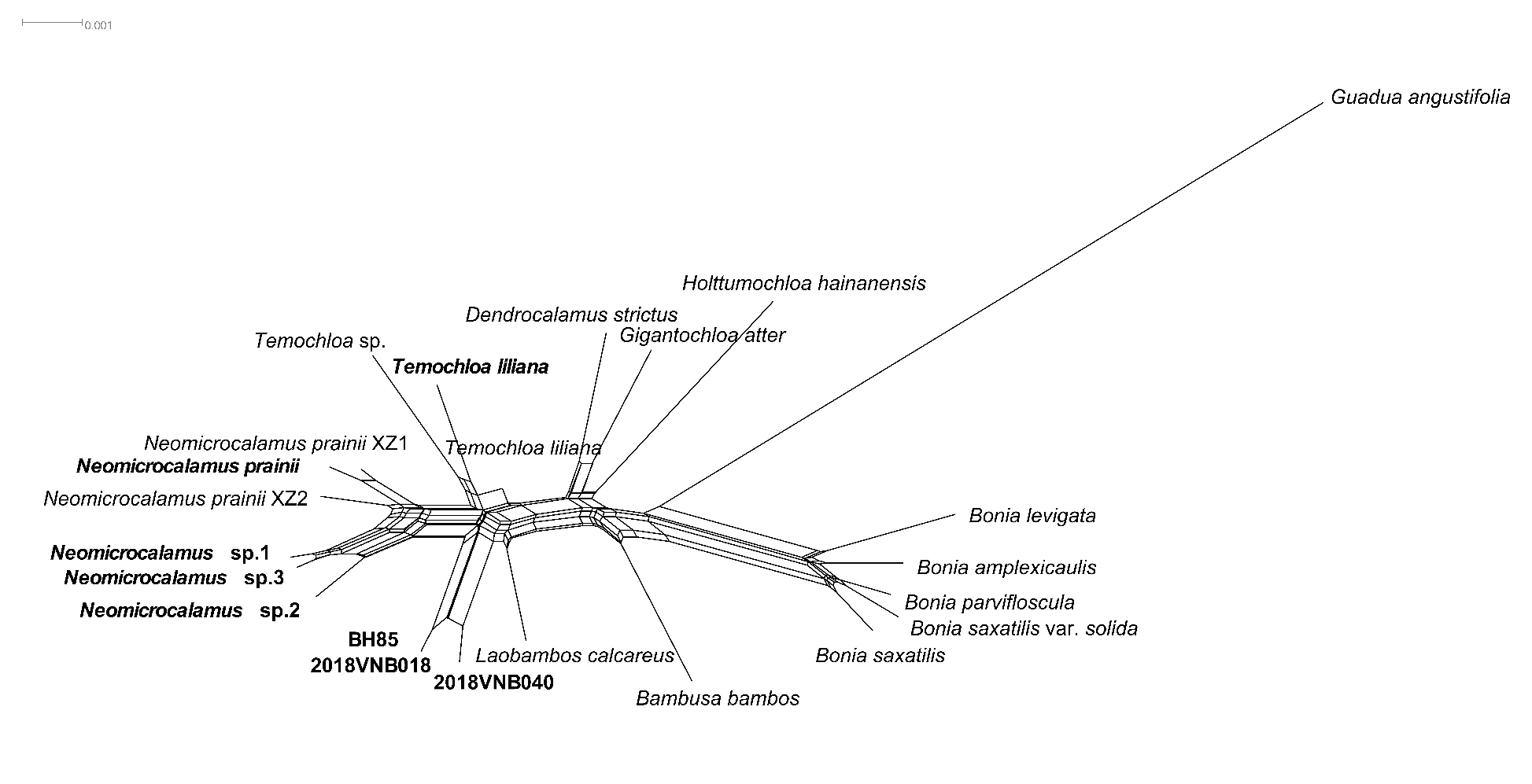
**

**Figure S1.** Network analysis of the plastid loci dataset of 23 samples. *BH85*, *2018VNB018* and *2018VNB040* represent the newly discovered bamboos; these and other accessions freshly obtained for this analysis are indicated in bold.
